# Similar Inflammatory Responses following Sprint Interval Training Performed in Hypoxia and Normoxia

**DOI:** 10.3389/fphys.2016.00332

**Published:** 2016-08-03

**Authors:** Alan J. Richardson, Rebecca L. Relf, Arron Saunders, Oliver R. Gibson

**Affiliations:** ^1^Environmental Extremes Lab, Centre for Sport and Exercise Science and Medicine, University of BrightonEastbourne, UK; ^2^Centre for Human Performance, Exercise, and Rehabilitation, Brunel University LondonUxbridge, UK

**Keywords:** high intensity training, altitude, endurance, inflammation, cytokine

## Abstract

Sprint interval training (SIT) is an efficient intervention capable of improving aerobic capacity and exercise performance. This experiment aimed to determine differences in training adaptations and the inflammatory responses following 2 weeks of SIT (30 s maximal work, 4 min recovery; 4–7 repetitions) performed in normoxia or hypoxia. Forty-two untrained participants [(mean ± *SD*), age 21 ±1 years, body mass 72.1 ±11.4 kg, and height 173 ±10 cm] were equally and randomly assigned to one of three groups; control (CONT; no training, *n* = 14), normoxic (NORM; SIT in FiO_2_: 0.21, *n* = 14), and normobaric hypoxic (HYP; SIT in FiO_2_: 0.15, *n* = 14). Participants completed a $\dot{\text{V}}{\text{O}}_{2\text{peak}}$ test, a time to exhaustion (TTE) trial (power = 80% $\dot{\text{V}}{\text{O}}_{2\text{peak}}$) and had hematological [hemoglobin (Hb), haematocrit (Hct)] and inflammatory markers [interleukin-6 (IL-6), tumor necrosis factor-α (TNFα)] measured in a resting state, pre and post SIT. $\dot{\text{V}}{\text{O}}_{2\text{peak}}$ (mL.kg^−1^.min^−1^) improved in HYP (+11.9%) and NORM (+9.8%), but not CON (+0.9%). Similarly TTE improved in HYP (+32.2%) and NORM (+33.0%), but not CON (+3.4%) whilst the power at the anaerobic threshold (AT; W.kg^−1^) also improved in HYP (+13.3%) and NORM (+8.0%), but not CON (–0.3%). AT (mL.kg^−1^.min^−1^) improved in HYP (+9.5%), but not NORM (+5%) or CON (–0.3%). No between group change occurred in 30 s sprint performance or Hb and Hct. IL-6 increased in HYP (+17.4%) and NORM (+20.1%), but not CON (+1.2%), respectively. TNF-α increased in HYP (+10.8%) NORM (+12.9%) and CON (+3.4%). SIT in HYP and NORM increased $\dot{\text{V}}{\text{O}}_{2\text{peak}}$, power at AT and TTE performance in untrained individuals, improvements in AT occurred only when SIT was performed in HYP. Increases in IL-6 and TNFα reflect a training induced inflammatory response to SIT; hypoxic conditions do not exacerbate this.

## Introduction

With a training volume and energy expenditure significantly less than traditional aerobic endurance training, sprint interval training (SIT) is considered a time-efficient method of improving cardiometabolic health (Gillen et al., 2016), skeletal muscle oxidative capacity and exercise performance (Gibala et al., 2006; Burgomaster et al., 2008). SIT is characterized by repeated bouts of exercise at a supramaximal intensity, interspersed by recovery periods (Burgomaster et al., 2005). This training induces a cascade of physiological adaptations, predominantly occurring at the muscle (metabolic adaptations), which can occur in as little as 2 weeks (Burgomaster et al., 2005; Gibala et al., 2006). Identified mechanisms facilitating improved exercise capacity via enhanced VO_2_ and O_2_ transport capacity include increased oxidative (Gibala et al., 2006) and glycolytic enzyme activity (Talanian et al., 2007; Daussin et al., 2008), muscle buffering capacity, glycogen content (Burgomaster et al., 2005) and increased skeletal muscle capillariation (De Smet et al., 2016; Montero and Lundby, 2016). Augmentation of exercise performance as a result of mitochondrial (Little et al., 2010) and vascular (Rakobowchuk et al., 2008) adaptations alongside improving hormonal responses (Kon et al., 2015), and insulin sensitivity (Richards et al., 2010), have reinforced SIT as a powerful training stimulus in diseased (Whyte et al., 2010), healthy untrained (Burgomaster et al., 2006; Gibala et al., 2006), and trained (Macpherson and Weston, 2015) populations.

Hypoxia has been widely observed as a potent stimuli for improving functional outcomes allied to exercise capacity (Rusko et al., 2004), with ascents ~2500 m identified as optimal (Chapman et al., 2014) using a LHTL model for improving endurance training. A number of review articles have supported the various applications for hypoxia in trained individuals (Wilber, 2007; Millet et al., 2010, 2013). Conversely, recent discussion has proposed a limited potential for the additional benefits of adding a hypoxic stimulus to training in trained populations (Lundby et al., 2012; McLean et al., 2014). This notion may however be, too broad to suggest hypoxia is entirely ineffective. Rather the benefits of the additional hypoxic stimulus likely elicit specific adaptations allied to the protocol which has been implemented, e.g., improved repeated sprint ability after repeated sprint training in hypoxia (Faiss et al., 2013b). It has been proposed that the addition of hypoxic stress during interval training is a mechanism to further enhance performance (Faiss et al., 2013a). The application of an additional stimuli to training (i.e., hypoxia) is challenging given the necessity to maintain an optimal training stimulus, e.g., training intensity (Millet and Faiss, 2012) and optimal level of altitude (Goods et al., 2014) for beneficial adaptations. Preliminary research supports the notion that performing SIT in hypoxia may enhance the magnitude of adaptation when compared to equivalent training prescription in normoxia (Puype et al., 2013). Mechanistically, SIT in hypoxia vs. normoxia provides additive stress resulting from an increased metabolic demand to exercise and increased relative stress during recovery thus potentiating greater adaptations (Buchheit et al., 2012). Training in hypoxia still maintains the favorable time efficiencies compared to traditional continuous lower intensity training, which makes the intervention favorable for a number of applications across populations (Gibala et al., 2012). The 30 s exercise duration of each bout of SIT requires a ~55% contribution from aerobic metabolism (Billaut and Bishop, 2009), this typically elicits greater performance detriments when training in hypoxia vs. normoxia, however prolonged recovery facilitates near complete recovery with the intention of maintaining sprint training specific stimuli (Millet and Faiss, 2012). This important balance between work:rest ratios theoretically preserves specific training stimuli associated with SIT, e.g., upregulated oxygen signaling genes and fast twitch fiber recruitment (Millet and Faiss, 2012), whilst increasing the metabolic disturbances required for adaptations to glycolytic pathways (Puype et al., 2013). Recent literature has identified that SIT in hypoxia augments adaptation during a 6 week training periods (Puype et al., 2013), however SIT in hypoxia over a 2 week training intervention may offer little additional benefit when compared to equivalent training in normoxia (Richardson and Gibson, 2015). Additional benefits of hypoxia have however also been shown in other repeated sprint training interventions of 2 (Faiss et al., 2015) to 4 weeks (Faiss et al., 2013b; Galvin et al., 2013; Kasai et al., 2015). This training modality, dose-response relationship remains to be fully determined in hypoxia, though it is known that 2 weeks is a sufficient time period to elicit adaptations in normoxia (Burgomaster et al., 2005, 2008).

Combinations of SIT and hypoxia induce significant physiological stress (Puype et al., 2013), which may impact recovery and therefore subsequent training or competition performance may be impaired (Goods et al., 2015). Interleukin-6 (IL-6) and tumor necrosis factor (TNFα) are pro-inflammatory cytokines both of which increase following equivalent training performed in hypoxia and normoxia (Svendsen et al., 2016). Plasma IL-6 increases with high intensity interval training (HIIT; Croft et al., 2009) and with increasing severity of hypoxia (Schobersberger et al., 2000; Turner et al., 2016), while TNFα appears to remain unchanged in response to passive hypoxic exposures (Turner et al., 2016). IL-6 has an important anti-inflammatory and adaptation-signaling role during the post-exercise recovery phase, with a greater increase in IL-6 post-hypoxic exercise reflective of a greater training stress (Fischer, 2006; Scheller et al., 2011). Given the failure for 2 weeks of SIT in hypoxia to elicit greater adaptations than SIT in normoxia (Richardson and Gibson, 2015) identification of the pro-inflammatory response to both interventions would facilitate greater understanding of the magnitude of additional training stimuli induced by hypoxia. Additionally, should a greater inflammatory response be identified, in the absence of improved adaptive response than equivalent training in normoxia, the use of SIT in hypoxia may be counterproductive.

The aims of this study were to investigate differences in the magnitude of training adaptations (VO_2max,_ time to exhaustion, Anaerobic Threshold) and inflammatory responses (IL-6 and TNFα), to 2 weeks of SIT performed in normoxia and hypoxia. It was hypothesized that due to the short 2-week (Richardson and Gibson, 2015), vs. longer 6 week (Puype et al., 2013), duration of the SIT, no differences in the magnitude of training adaptations would be observed. Additionally it was hypothesized that SIT performed in hypoxia will elicit greater inflammatory responses than SIT performed in normoxia due to an increased physiological stress caused by an inhibited aerobic contribution during recovery.

## Methods

### Subjects

Forty-two untrained, but recreationally active individuals (27 males, 15 females) age 21 ± 1 years, body mass 72.1 ± 11.4 kg, and height 173 ± 10 cm volunteered to take part in this experiment (Table 1). No differences in anthropometric or fitness measures were found between groups (*p* > 0.05). Participants were informed of the procedures to be employed in the study and associated risks, which had the approval of the University of Brighton Research Ethics Committee (ESREGC/06/14). All participants provided written, informed consent. The participants were non-smokers and had not spent time above 2000 m in the 2 months prior to the study. Participants were advised to refrain from alcohol and caffeine for 24 h prior to testing and to maintain their normal unstructured training habits (<2 hr.wk) throughout the study.

**Table 1:** **Participant baseline values for anthropometric and aerobic capacity measures**.

	**CONT**	**NORM**	**HYP**
Body Mass (Kg)	70.3 ± 13	73.3 ± 11	72.5 ± 10
Height (cm)	172 ± 10	174 ± 11	174 ± 8
Age (years)	20 ± 1	20 ± 1	20 ± 1
Hb (g.dL^−1^)	14.5 ± 1.4	14.2 ± 1.5	14.6 ± 1.8
Hct (%)	44 ± 2	45 ± 2	44 ± 2
TTE (s)	606 ± 280	589 ± 372	633 ± 330
$\dot{\text{V}}{\text{O}}_{2\text{peak}}$ (mL.kg^−1^.min^−1^)	42.1 ± 9.7	42.2 ± 8.6	43.6 ± 7.9

*Hb, B-Hemoglobin; Hct, Hematocrit; TTE - Time to Exhaustion*.

### Experimental design

The 42 participants were randomly assigned and equally split for number (*n* = 14) and sex (9 males, 5 females), to one of the three intervention groups; a normoxic (NORM) (FiO_2_: 0.2093) environment, a moderate normobaric hypoxic (HYP) (FiO_2_: 0.15, range 0.148–0.152; FiCO_2_: 0.0008, range 0.0003–0.0028) environment and a control (CONT) normoxic non-training group (Table 1). All testing was performed in a nitrogen enriched normobaric hypoxic chamber with temperature (19°C) and humidity (40%) regulated by air conditioning (Altitude Centre, London, UK).

**Figure 1:**
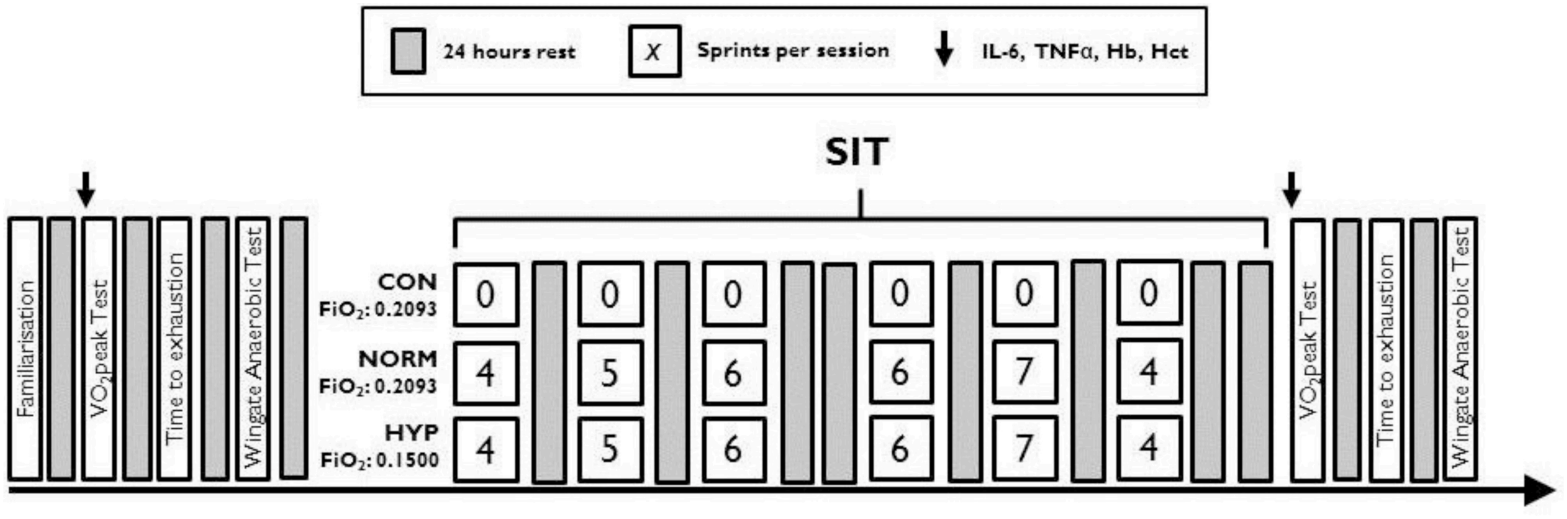
**Schematic of the testing and sprint interval training protocol for each training group**.

Familiarization of the Wingate anaerobic test (WAnT) and time to exhaustion (TTE) was performed prior to any of the experimental testing. Preliminary testing involved participants completing in sequence, a peak oxygen consumption ($\dot{\text{V}}{\text{O}}_{2\text{peak}}$) incremental test, a time to exhaustion cycle test (TTE) and a Wingate anaerobic test (WAnT), with 24 h separating each test. Prior to each $\dot{\text{V}}{\text{O}}_{2\text{peak}}$ test, venous blood was taken to measure haematocrit (Hct), hemoglobin (Hb), Interleukin- 6 (IL-6), and TNFα, see Figure 1. The SIT consisted of six WAnT sessions over a 2-week period with 24–48 h between each session (Figure 1). Each training session followed that an established SIT protocol (Burgomaster et al., 2005), consisting of an increasing number of WAnTs [four to seven 30 s “all out” efforts on a cycle ergometer interspersed with 4 min warm up/recovery (60 W)]. Throughout training heart rate [HR, bts.min^−1^ (Polar FT1, Polar Electro, Kempele, Finland)], peripheral arterial oxygen saturation [SpO_2,_ % (PalmSat 2500, Nonin Medical Inc., Minnesota, USA)], and rating of perceived exertion [RPE; Borg Scale 6–20 (Borg, 1982)] were measured immediately after each WAnT and every minute thereafter during recovery. Those in the CONT group maintained usual physical activity regimes for the 2 week period. Forty-eight hours after the final SIT session all participants repeated the $\dot{\text{V}}{\text{O}}_{2\text{peak}}$, TTE and WAnT protocols, each separated by 24 h.

### Preliminary and post testing

Participants performed an incremental test to volitional exhaustion on an electromagnetically-braked cycle (Schoberer Rad Messtechnik equipped with 8 strain gauges, SRM, Germany), with the zero offset calibration procedure performed on the SRM PowerMeter prior to each test, to determine $\dot{\text{V}}{\text{O}}_{2\text{peak}}$. Starting at 100 W, there was a stepwise increase in power of 20 W.min^−1^. Expired gases were obtained using a breath by breath gas analyser (Metamax 3X, Cortex, Germany). HR and RPE were taken at the end of every stage. Anaerobic threshold was computer-determined with additional visual inspection to determine the first breakpoint in ventilatory parameters.

Twenty-four hours later, participants performed a TTE at an intensity corresponding to 80% peak power output (PPO) attained during the pre-SIT assessment of $\dot{\text{V}}{\text{O}}_{2\text{peak}}$, on a cycle ergometer at a target cadence of 80 revs.min^−1^ (Monark, model 864, Sweden). The test was terminated at volitional exhaustion when the participants' cadence fell below 40 revs^.^min^−1^; exercise duration (seconds) was then determined. Capillary blood was collected from the fingertip pre and 2 min post TTE for analysis of blood lactate (2300 Stat Plus, YSI Life Sciences, USA).

Prior to each $\dot{\text{V}}{\text{O}}_{2\text{peak}}$ test 10 mL of blood was collected from the ante-cubital fossa. Whole blood (~50 μL) was divided into two heparinised capillary tubes (Hawksley & Sons Ltd., England) then centrifuged (Hematospin 1300, Hawksley & Sons Ltd., England) at 1000 rpm for 1.5 min to calculate the haematocrit using a micro haematocrit reader (Hawksley & Sons Ltd., England); the average of duplicate samples was recorded. Hemoglobin concentration (B-Hemoglobin Photometer, Hemocue, Sweden) was determined via the average of triplicate samples (B-Hemoglobin Microvettes, Hemocue, Sweden). The remaining blood was transferred into two 5 mL EDTA tubes and centrifuged at 5000 rpm for 10 min. Plasma was then extracted into microvettes and stored at –86°C. IL-6 and TNFα concentrations were analyzed using Enzyme-linked immunosorbent assays in accordance with manufacturer instructions (DuoSet ELISA Development System; R&D Systems Inc., Abingdon, UK) with corrections made for the change in plasma volume (Dill and Costill, 1974). The technical error of measurement (TEM) between duplicate samples for IL-6 was 7.1%, with a unit error value of 2.76 pg.mL^−1^ and for TNFα it was 4.1%, with a unit error value of 518.7 pg.mL^−1^.

### WAnT

Each WAnT, performed 24 h following the TTE, consisted of 30 s of “all out” maximal cycling on a friction-loaded cycle ergometer (Monark Ergomedic Peak Bike 894e, Monark Exercise AB, Sweden). The load was calculated as 7.5% body mass [0.075 kg/kg.bm^−1^, (Bar-Or, 1987)]. The onset of each sprint was marked with a “3-2-1. GO!” countdown, and participants were instructed to cycle for ~2 s against the ergometers inertial resistance before the full load was released at 70 rpm. Participants were required to stay seated on the saddle and were verbally encouraged throughout the test. Each sprint was followed by a 4 min recovery period—participants were required actively recover (unloaded cycling at 60 revs.min^−1^). Peak power output, average 30-s power output (total work), and fatigue index [also known as rate of power decline; ((Peak Power Output–Min Power Output)/Peak Power Output) × 100) (%)] were recorded by Monark Anaerobic Test software (ver. 3.2.7.0, Monark Exercise AB, Sweden).

### Sprint interval training

All SIT was performed on a cycle ergometer (Monark, model 864, Sweden) against a resistance of 0.075 kg.kg^−1^ body mass, from a rolling start of 70 revs·min^−1^. Participants were verbally encouraged throughout. The sprints were interspersed with a 4 min active recovery period of cycling at 60 W. Power measures were recorded using Monark Anaerobic Test software (Monark, Sweden) continuously throughout the sprints. The number of sprints increased from four to seven over the 2 weeks (total six sessions). Training days were interspersed with one rest day (Figure 1). SpO_2_ and HR were monitored using a finger pulse oximeter (Nonin 2500, Nonin Medical Inc., USA) 1 min after every sprint.

### Statistical analysis

Data were tested for normality, skewness and kurtosis. Data were normally distributed unless otherwise stated. A Two Way Mixed Design ANOVA was performed separately on each of the independent variables; $\dot{\text{V}}{\text{O}}_{2\text{peak}}$, TTE, Peak Power, Mean Power, Fatigue index (from WAnT test), IL-6, TNFα, Hb, and Hct, to determine whether there was a significant change between the three conditions (HYP, NORM, and CON) over two time-points (pre and post). The mean sessional recovery HR and SpO_2_ observed following each SIT were analyzed using a mixed 2-way ANOVA using the Greenhouse-Geisser correction to determine whether there was a significant change between the two training conditions (HYP and NORM) over the six SIT sessions. Adjusted Bonferroni comparisons were used as *post-hoc* analyses for all ANOVA. Partial eta squared was used to calculate effect sizes (*np*^2^; small = 0.01, medium = 0.06, large = 0.13) were calculated to analyse the magnitude and trends with data. All data were reported as Mean ± *SD*. All statistical tests followed a significance level of *p* < 0.05. The statistical package used was SPSS (SPSS Inc., Chicago, USA, version 20.0).

## Results

### Endurance capacity

**Figure 2:**
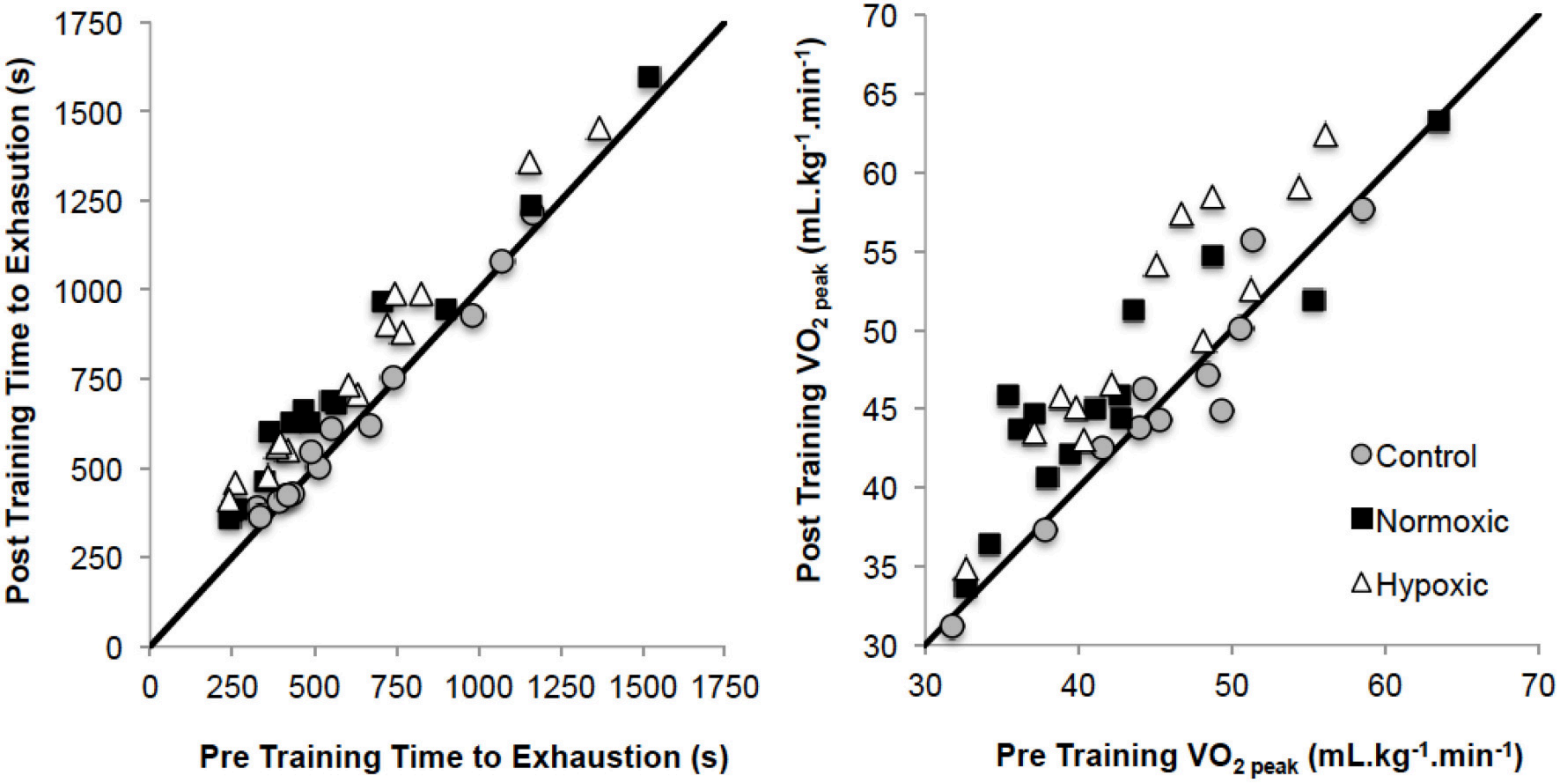
**Pre training to post training time to exhaustion and V°O_**2peak**_ changes for the three training groups**. Solid black lines demonstrates the line of equality.

$\dot{\text{V}}{\text{O}}_{2\text{peak}}$ was significantly different from pre to post-training (*p* = 0.001, *np*^2^ = 0.44), and between different training groups (*p* = 0.002, *np*^2^ = 0.28, Figure 2). *Post-hoc* analysis observed increases in HYP (*p* = 0.001; +0.39 ± 0.29 L.min^−1^; 43.6 ± 8.0 to 48.8 ± 9.2 mL.kg^−1^.min^−1^) and NORM (*p* = 0.002; +0.32 ± 0.38 L.min^−1^; 42.2 ± 8.6 to 46.0 ± 7.5 mL.kg^−1^.min^−1^), but not for CONT (*p* = 0.906; 42.1 ± 9.7 to 42.2 ± 9.7 mL.kg^−1^.min^−1^). Relative power at $\dot{\text{V}}{\text{O}}_{2\text{peak}}$ (W.kg^−1^) was greater pre to post (*p* = 0.001, *np*^2^ = 0.42) overall and for the pre-post*group interaction (*p* = 0.004, *np*^2^ = 0.25). *Post-hoc* analysis observed increases in HYP (*p* = 0.001; 3.90 ± 0.72 to 4.20 ± 0.80 W.kg^−1^) and NORM (*p* = 0.001; 3.54 ± 0.73 to 3.86 ± 0.86 W.kg^−1^), but not for CONT (*p* = 0.872; 3.75 ± 0.56 to 3.76 ± 0.70 W.kg^−1^).

The anaerobic threshold (AT) increased pre to post SIT (*p* = 0.001, *np*^2^ = 0.30) overall and for the pre-post*group interaction (*p* = 0.001, *np*^2^ = 0.29). *Post-hoc* analysis only observed increases in HYP (*p* = 0.001; 22.6 ± 4.1 to 24.6 ± 4.2 mL.kg^−1^.min^−1^) and not NORM (*p* = 0.050; 22.9 ± 4.7 to 24.0 ± 4.8 mL.kg^−1^.min^−1^) or CONT (*p* = 0.417; 22.7 ± 5.6 to 22.4 ± 4.9 mL.kg^−1^.min^−1^). Relative power at AT was greater pre to post-training (*p* = 0.001, *np*^2^ = 0.313) overall and for the pre-post*group interaction (*p* = 0.006, *np*^2^ = 0.23). *Post-hoc* analysis observed increases in both HYP (*p* = 0.001; 2.00 ± 0.36 to 2.26 ± 0.45 W.kg^−1^) and NORM (*p* = 0.017; 1.88 ± 0.40 to 2.04 ± 0.49 W.kg^−1^) but no change in CONT (*p* = 0.925; 1.98 ± 0.35 to 1.97 ± 0.37 W.kg^−1^).

TTE was significantly different from pre to post test (*p* = 0.001, *np*^2^ = 0.82) overall, and for the pre-post*group interaction (*p* = 0.001, *np*^2^ = 0.64, Figure 2). *Post-hoc* analysis observed increases in HYP (*p* = 0.001; 633 ± 330 to 787 ± 326 s) and NORM (*p* = 0.001; 589 ± 373 to 729 ± 351 s), but not for CONT (*p* = 0.212; 607 ± 280 to 620 ± 274 s).

### Power capacity during WAnT

Peak power was not different during the WAnT from pre to post-training (*p* = 0.530, *np*^2^ = 0.010) or between different training groups (CONT; 11.2 ± 3.0 to 10.9 ± 2.2 W.kg^−1^, NORM; 11.0 ± 1.3 to 11.4 ± 1.4 W.kg^−1^, HYP; 11.7 ± 2.4 to 11.9 ± 2.4 W.kg^−1^) (*p* = 0.052, *np*^2^ = 0.141).

Similarly, mean power was not different from pre to post-training (*p* = 0.653, *np*^2^ = 0.005), or between training groups (CONT; 3.8 ± 0.8 to 3.7 ± 0.8 W.kg^−1^, NORM; 4.0 ± 1.1 to 4.1 ± 0.9 W.kg^−1^, HYP; 3.6 ± 1.3 to 3.8 ± 1.2 W.kg^−1^) (*p* = 0.319, *np*^2^ = 0.057).

Fatigue Index was found to be significantly reduced from pre to post-training *(p* = 0.001, *np*^2^ = 0.968), although there was no significant difference between training groups CONT; 62.7 ± 10.7 to 62.9 ± 12.3%, NORM; 63.5 ± 9.2 to 61.8 ± 9.4%, HYP; 65.1 ± 12.6 to 62.3 ± 10.1%) (*p* = 0.851, *np*^2^ = 0.008).

### Hematological and inflammatory markers

Hb was significantly different from pre to post-training (*p* = 0.036, *np*^2^ = 0.11). However, this increase was not different between training groups (*p* = 0.082, *np*^2^ = 0.12) for CONT (14.6 ± 1.5 to 14.6 ± 1.5 g.dL^−1^), NORM (14.3 ± 1.5 to 14.3 ± 1.3 g.dL^−1^), and HYP (14.7 ± 1.8 to 15.0 ± 1.7 g.dL^−1^).

Hct was not different from pre to post-training (*p* = 0.701, *np*^2^ = 0.00) or between groups (*p* = 0.215, *np*^2^ = 0.08) for CONT (44.0 ± 2.8 to 43.7 ± 2.4%), NORM (44.9 ± 2.1 to 45.0 ± 2.7%), and HYP (45.7 ± 2.8 to 46.0 ± 3.1%).

Blood lactate increased in all TTE tests (*p* = 0.001, *np*^2^ = 0.29). Post TTE blood lactate values increased significantly with NORM (*p* = 0.01 5.07 ± 0.77 to 5.62 ± 1.01 mmol.L^−1^), and HYP training (*p* = 0.010; 5.24 ± 0.9 to 5.76 ± 0.82 mmol.L^−1^), but not in CONT (*p* = 0.101; 6.42 ± 0.8 to 6.52 ± 0.95 mmol.L^−1^).

IL-6 was significantly different from pre to post test (*p* = 0.001, *np*^2^ = 0.39) and this increase was different between training groups (*p* = 0.007, *np*^2^ = 0.23). *Post-hoc* analysis observed increases for HYP (*p* = 0.001; 1.7 ± 0.2 to 2.0 ± 0.2 pg.mL^−1^) and NORM (*p* = 0.003; 1.7 ± 0.2 to 2.0 ± 0.3 pg.mL^−1^), but not for CONT (*p* = 0.836; 1.7 ± 0.2 to 1.7 ± 0.2 pg.mL^−1^).

TNFα was significantly different from pre to post test (*p* = 0.006, *np*^2^ = 0.175), however, was not significantly different between training groups (*p* = 0.151, *np*^2^ = 0.09) (NORM; 3.0 ± 0.6 to 3.4 ± 0.9 pg.mL^−1^; HYP; 3.1 ± 0.8 to 3.3 ± 0.6 pg.mL^−1^; CONT; 2.7 ± 0.7 to 2.8 ± 0.6 pg.mL^−1^).

### Training markers

**Figure 3:**
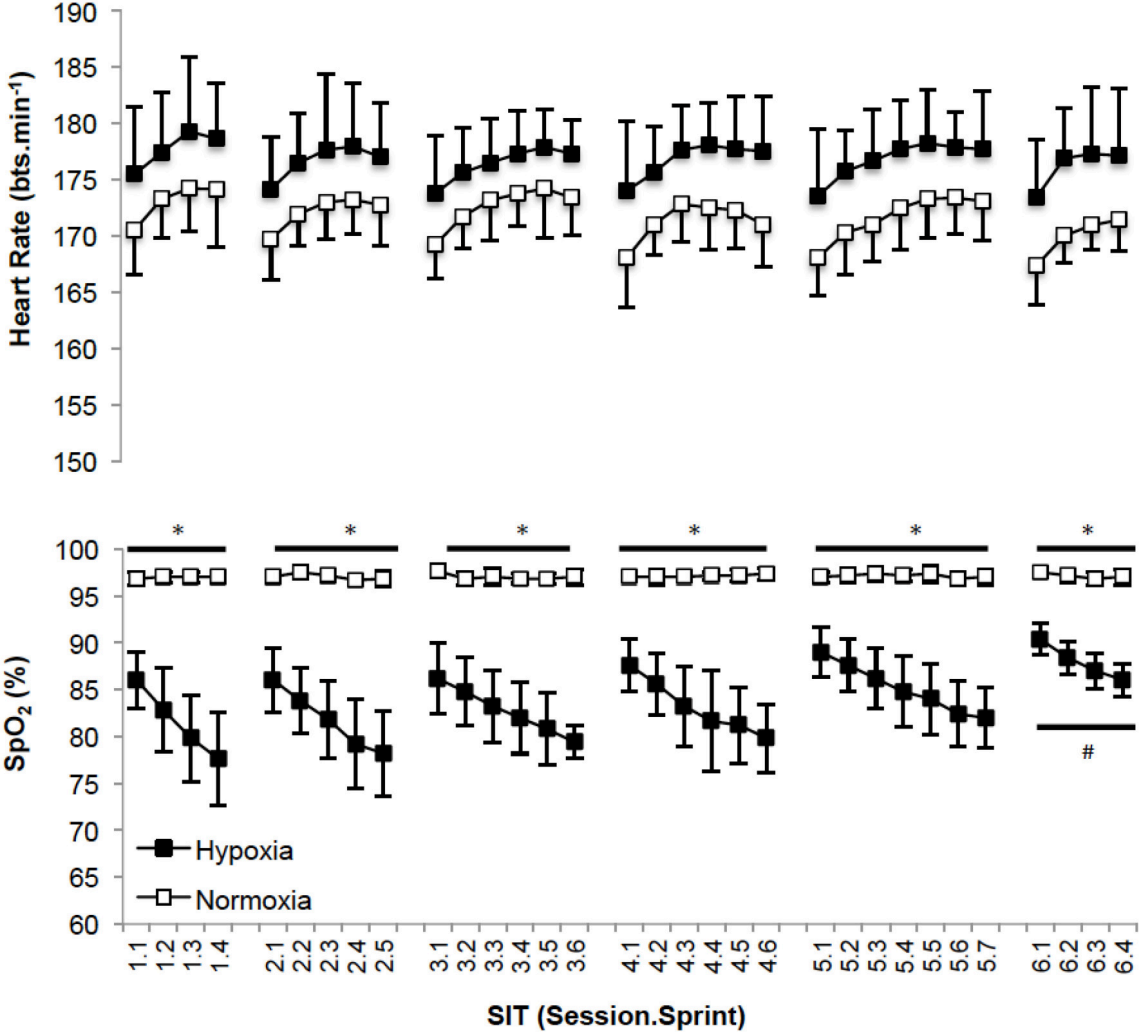
**(Mean ± ***SD***) SpO_**2**_ and heart rate values after each sprint for all training sessions**. ^*^Denotes significant difference (*p* < 0.05) between conditions within session. #Denotes significant difference (*p* < 0.05) from first, second, third, fourth and fifth sessions.

Recovery HR was not different between sessions (*p* = 0.250; *np*^2^ = 0.11) or for the different training groups (*p* = 0.420; *np*^2^ = 0.04, Figure 3). SpO_2_ was different between sessions (*p* = 0.001; *np*^2^ = 0.30) and significantly less with HYP training (*p* = 0.001; *np*^2^ = 0.20). *Post-hoc* analysis of SpO_2_ is presented in Figure 3 for clarity.

## Discussion

The aim of this experiment was to quantify the improvements in aerobic capacity and aerobic performance following 2 weeks of SIT in normoxia, and hypoxia in comparison to a non-trained control group. In support of previous work in the field, we have demonstrated that SIT improved V°O_2peak,_ power at AT and TTE to comparable magnitude associated with interval training in normoxia (Burgomaster et al., 2005; Gibala et al., 2006; Hazell et al., 2010), and hypoxia (Galvin et al., 2013; Puype et al., 2013; Gatterer et al., 2014; Brocherie et al., 2015; Richardson and Gibson, 2015; De Smet et al., 2016). In contrast to our hypothesis, an additive effect of performing SIT in hypoxia vs. normoxia was observed with regards to the AT, with no changes observed in NORM and CONT. Equality of increases in IL-6 48 h following normoxic and hypoxic SIT, also opposed our initial hypothesis. This change in concentration reflecting a training induced inflammatory response absent in controls, but with no greater, undesirable inflammatory response observed in hypoxia.

### Adaptations to aerobic capacity and exercise performance

In the present study $\dot{\text{V}}{\text{O}}_{2\text{peak}}$, TTE, and power at the AT increased following SIT in HYP and NORM suggesting improved oxidative phosphorylation had occurred (Burgomaster et al., 2005, 2006). Interestingly, when considering AT expressed at mL.kg^−1^.min^−1^ this adaptation only occurred in HYP (+9.5%), not NORM (+5.0%). Puype et al. (2013) acknowledged that their 6-week intervention improved the power corresponding to the AT (quantified from a 4 mmol.L^−1^ lactate concentration) by ~7% (in normoxia) and 9% (in hypoxia) only following hypoxic, and not normoxic training. This adaptation was associated with an increase in muscle phosphofructokinase (PFK; hypoxia = +59%, normoxia = +17%). It was reported that VO_2max_ increased (hypoxia = +7.4%; normoxia = +5.8%) and TTE improved (hypoxia = +5.0%; normoxia = +2.9%) compared to controls, but with no difference when performing the training in hypoxia vs. normoxia (Puype et al., 2013). These observations are in line with our experiment. Our data is also supportive of an increased AT following SIT (Puype et al., 2013), and therefore increased glycolytic capacity via elevated PFK. Methodological differences in identifying the threshold, and the three-fold protocol duration of Puype et al. (2013) provide a rationale for the contrast between significant changes in the power at the AT following training in hypoxia (Puype et al., 2013) and the absence of a further improvement in power at the AT in comparison to normoxia as observed by ourselves. The improved AT (mL.kg^−1^.min^−1^) following SIT in hypoxia in comparison to SIT in normoxia in our study may have also been acknowledged by Puype et al. (2013), unfortunately this was not quantified. SIT, or similar training, elicits known metabolic adaptations, e.g., increased oxidative (Gibala et al., 2006) and glycolytic enzyme activity (Talanian et al., 2007; Daussin et al., 2008), improved muscle buffering capacity, elevated intramuscular glycogen content (Burgomaster et al., 2005) and increased skeletal muscle capillarisation (Puype et al., 2013). These are likely induced from the performance of the sprints within the protocol, with hypoxia potentiating greater metabolic disturbances in vs. normoxia (Faiss et al., 2013b; Galvin et al., 2013; Puype et al., 2013). Even greater improvements in glycolytic function may have been identified by increased mean power and improved fatigue index during the WaNT (Puype et al., 2013), however this was not case suggesting the mode of training was not structured effectively to facilitate this. Increased lactate in response to improved TTE suggests a greater tolerance to metabolic acidosis via group III/IV afferents (Amann et al., 2015) with SIT not demonstrating improved lactate clearance as supported by evidence elsewhere (Juel et al., 2004). The absence of a difference in peak and mean power, and fatigue index during the WAnT demonstrate that SIT in normoxia and hypoxia was effective at improving aerobic metabolic pathways. Mechanisms supporting the physiological ($\dot{\text{V}}{\text{O}}_{2\text{peak}}$) and performance (TTE) responses in our normobaric hypoxic environment do not appear hematological given the lack of intragroup difference in Hb and Hct (Table 2), this is in agreement with the proposal of others (Richalet and Gore, 2008), reinforcing the metabolic adaptive pathway. The measurement of Hb and Hct would however be improved by assessing total Hb mass thus accounting for changes otherwise lost when measuring the concentration (Burge and Skinner, 1995; Schmidt and Prommer, 2005). Based upon the expected lack of hematological adaptations between groups, aforementioned modulators of oxygen utilization at the muscle are most likely improved by SIT irrespective of the FiO_2_ in which it is performed. Interestingly, improvements in SpO_2_ were observed within the HYP group by the 6th session (Figure 3). This suggests some level of acclimation to hypoxia had occurred. SIT may therefore be effective at mitigating desaturation known to occur during repeated/intermittent sprint performance in hypoxia (Bowtell et al., 2014; Turner et al., 2014). Accordingly, future work investigating training adaptations to SIT could consider the benefits of hypoxic SIT to prepare athletes, rather than untrained individuals as in the present experiment, for competition in hypoxia (Girard et al., 2013; Millet et al., 2013). Concurrent mechanistic work may also wish to consider whether our equal pre to post-intervention measures of Hb and Hct data confirms a lack of hematological adaptation i.e., HB_mass_, in favor of improved metabolic pathways (Faiss et al., 2013b). Additionally exploration of the mechanisms by which preservation of SpO_2_ occurs in response to SIT in hypoxia may also be considered. With no clear additional benefit of performing 2 weeks of SIT in hypoxia rather than normoxia (on $\dot{\text{V}}{\text{O}}_{2\text{peak}}$, and TTE performance lasting ~10 min, Table 2), implementing this training appears largely unreasoned from a performance perspective at the present time, particularly given the challenge of facilitating training in this environment.

**Table 2:** **Change (%) in aerobic capacity, time to exhaustion (TTE), bloods and inflammatory measures in each group**.

	**CONT**	**NORM**	**HYP**
$\dot{\text{V}}{\text{O}}_{2\text{peak}}$	0.9 ± 11.4	9.8 ± 9.4^*^	11.9 ± 6.7^*^
Power at $\dot{\text{V}}{\text{O}}_{2\text{peak}}$	−0.1 ± 5.9	8.8 ± 7.8^*^	7.7 ± 6.0^*^
AT	−0.4 ± 4.4	5.0 ± 8.2	9.5 ± 7.1^*^
Power at AT	−0.3 ± 12.4	8.0 ± 10.2^*^	13.3 ± 8.5^*^
TTE	3.4 ± 7.3	32.3 ± 19.0^*^	32.2 ± 20.7^*^
WAnT Peak Power	−1.7 ± 7.0	3.6 ± 3.7	1.8 ± 5.9
WAnT Mean Power	−2.4 ± 5.4	3.1 ± 9.3	4.2 ± 10.6
WAnT Fatigue Index	0.5 ± 11.4	−2.6 ± 4.7	−3.4 ± 9.5
IL-6	1.2 ± 11.8	20.1 ± 22.1^*^	17.4 ± 15.3^*^
TNFα	3.5 ± 17.5	12.9 ± 16.9^*^	10.8 ± 16.3^*^
Hb	0.1 ± 1.6	0.7 ± 4.3	2.7 ± 3.0
Hct	−0.7 ± 2.0	0.4 ± 2.7	0.7 ± 1.9

*AT, Anaerobic Threshold; TTE, Time to Exhaustion; WAnT, Wingate Anaerobic Test; IL-6, Interleukin 6; TNFα, Tumor Necrosis Factor; Hb, Hemoglobin; Hct, Hematocrit*.

* *Denotes significant change with training*.

### Inflammatory responses to SIT in normoxia and hypoxia

The increase in basal IL-6 following both normoxic and hypoxic SIT (Table 2) reflects the intensity and duration of the activity as widely observed elsewhere (Fischer, 2006). The functional significance of an increased IL-6 is complex (Gleeson and Bishop, 2000), with increased exercise requirements (Croft et al., 2009) and increasing metabolic stress, e.g., via hypoxia in isolation, or hypoxia related increases in the relative exercise intensity (Schobersberger et al., 2000) typically eliciting larger responses. IL-6 has an important anti-inflammatory and adaptation signaling role during the post-exercise recovery phase (Svendsen et al., 2016), with a greater increase in IL-6 post-hypoxic exercise reflective of a greater training stress (Fischer, 2006; Scheller et al., 2011). A reduction of IL-6 is a known training adaptation (Fischer, 2006); the elevation of the cytokine 48 h following the final training session however indicates that recovery/adaptation was incomplete. Irrespective of the consequential effects of increased basal IL-6, the current data appeases concerns that training in hypoxia as having an impairment upon individuals when compared to equivalent normoxic training. The increase in TNF-α alongside IL-6 is similar to other data demonstrating relationships between these inflammatory biomarkers and exercise (Gleeson and Bishop, 2000). Interestingly, a similar magnitude of inflammatory response to SIT was observed for TNFα as IL-6, however this was not statistically different to controls despite the ~12% difference between NORM and HYP, and CON. This disparity between IL-6 and TNFα may be due to the greater within group variability observed with this inflammatory marker (~25%) or the lack of hypoxia specific TNFα induction during passive (Turner et al., 2016) or active (Svendsen et al., 2016) exposures. Nonetheless performing SIT in hypoxia did not exacerbate the inflammatory response in comparison to normoxia, and is therefore unlikely to be detrimental to subsequent training or performance. A more precise quantification of training load within each group, and subsequently ensuring equality of load between groups, would give further confidence in the equality of inflammatory responses to SIT performed in either normoxia or hypoxia. Should absolute or relative internal/external training load be different between SIT performed in normoxia or hypoxia, then this may influence the interpretation of the inflammatory markers and suggest that hypoxia reduces or exacerbates the responses.

Similar basal inflammatory markers IL-6 and TNFα 24 h post the final SIT suggests that the increased stress of training in hypoxia is equal to that of normoxia and would not be detrimental to the individual. This equality of cytokine response is in agreement with the comparable magnitude of increases in IL-6 (hypoxia = +57%, normoxia = +56%) and lack of change in TNFα 2 h after a 75 min submaximal cycle in either condition (Svendsen et al., 2016). Accordingly the benefits of hypoxic SIT to prepare athletes for competition in hypoxia (Millet et al., 2013) can be determined at least equal to that of equivalent training in normoxia, without further compromising subsequent activities. Given the abundance of cellular and molecular pathways associated with SIT and HIIT, and hypoxia, this experiment presents a constrained overview of the responses. To optimize the application of SIT in hypoxia, future work should consider measurement of a wider spectrum of blood and muscle markers of training adaptations associated with SIT, and hypoxia both in isolation, and in combination. Additionally, further analysis of the impact of SIT in hypoxia on stress markers should be considered at a basal level (as determined within the present experiment) but also regarding the kinetics of a within session increase and the subsequent time-course to return to baseline prior to, and beyond our 48 h measurement point.

## Conclusion

Two weeks of SIT in hypoxia improves peak oxygen uptake, time to exhaustion and power at the anaerobic threshold, to a similar magnitude as equivalent training in normoxia. Improvements in the anaerobic threshold itself were only elicited in response to SIT in hypoxia, and not normoxia, highlighting the additional benefit of training in this environment. Equality of increases in basal IL-6 and TNFα following SIT in hypoxia and normoxia suggests that hypoxia does not exacerbate inflammatory processes.

## Author contributions

AR, RR, AS, and OG conceived and design the experiment. RR and AS performed the data collection. AR, RR, AS, and OG performed the statistical analysis and interpretation of data. AR, RR, AS, and OG participated in drafting the article or revising it critically for important intellectual content. AR, RR, AS, and OG approved the final manuscript.

### Conflict of interest statement

The authors declare that the research was conducted in the absence of any commercial or financial relationships that could be construed as a potential conflict of interest. The reviewer RF and handling Editor declared their shared affiliation, and the handling Editor states that the process nevertheless met the standards of a fair and objective review.
